# Public Health Department

**Published:** 1900-03

**Authors:** I. J. Jones

**Affiliations:** Secretary Quarantine Department


### Public Health Department.

I. J. JONES, M. D., SECRETARY QUARANTINE DEPARTMENT.

The State Health Officer has issued the following circular to all the railroad managers of the State:

"Dear Sir: I regret to state that smallpox has made considerable progress in various parts of the State, and chiefly along railroad lines. In many instances it has been introduced in the persons of railroad employes, practically in the persons of laboring men. In view of these facts, I earnestly request that you will issue strict instructions to all your agents and foremen to be very careful in this, and to promptly report any eruptive sickness among their men.

"I will also urge that you order the vaccination of every person, whomsoever, in your employment, at the earliest practicable moment. This should be applied to all persons who have not been successfully vaccinated in the last three years. These measures, if enforced, will be of the greatest value to the public health of the State, and highly appreciated by me.

"W. F. Blunt, State Health Officer."

Favorable replies to this circular have been received from the following railroads: Fort Worth & Rio Grande; Kansas City, Pittsburg & Gulf; Pecos Valley & Northeastern; Fort Worth & Denver City; Austin & Northwestern; International & Great Northern; Texas Midland; Texas-Mexican; St. Louis & Southwestern; Rio Grande & El Paso; Gulf, Colorado & Santa Fe; Gulf, Beaumont & Kansas City; Chicago, Rock Island & Texas; Cane Belt; Texas & Pacific; Sherman, Shreveport & Southern; Houston & Texas Central, and Southern Pacific.

---

On the 28th day of February the Surgeon-General of the Marine Hospital Service informed the State Health Officer of Texas of the appearance of bubonic plague in the Island of Azumel, State of Yucatan, Mexico. Quarantine was immediately ordered against this place at all the Rio Grande and Gulf Coast stations. This disease has steadily spread for the last two years, until now the continent of North America is alone unaffected, and several ships with the disease aboard have recently reached our western shores.

---

As a result of recent observation of many cases of disputed diagnosis of smallpox, and on account of the mild type of these cases, as they are usually presented, the editor of this department submits the following diagnosis table, prefacing it by saying that it is compiled from recent authorities, though arranged to suit conditions now prevailing:

DIFFERENTIAL DIAGNOSIS OF SMALLPOX.

| SMALLPOX. | CHICKEN POX. |
|---|---|
| Affects all ages alike. | A disease of children. Many authorities assert that it never occurs in adults. |
| Modified and prevented by vaccination. | Not affected by vaccination. |
| Icubation eight to twenty days. | Icubation ten to fourteen days. |
| Onset usually by chill, followed by temperature of 103 degrees to 105 degrees. Headache, backache, boneache, nausea and vomiting. | Chill unusual. Temperature rarely ever exceeds 103 degrees. Headache, etc., not marked. |
| Eruption appears on third day, and marked by sudden fall of temperature to normal. | Eruption appears in a few hours, and fever continues throughout eruptive stage. |

| | |
|---|---|
| Initial rash occurs in 13 per cent. of cases, and is situated in groin, inside of thighs, and auxilary regions, and regions affected by it are exempt from subsequent eruption. | No initial rash. |
| Papular eruption appears on third day. | Primary eruption in form of irregular blotches in a few hours after initial fever. |
| Appears, in very large majority of cases, first on forehead and around mouth. | Usually appears on back and trunk. |
| Extends over entire body within twenty-four hours, except in very mild cases. | Rarely ever extends over entire body. |
| Eruptions give indurated or "shotty feel," as soon as they appear, and even before they are perceptible on surface. | Eruption is not indurated, nor raised above surface. |
| Eruption most numerous and conspicuous on exposed surfaces of body, as the face, neck and hands. | Eruption principally confined to trunk, and very little except on protected parts of body. |
| Eruption on palms of hands and soles of feet. | Eruption rarely ever occupies these sites. |
| Papules become vesicular on fourth and fifth days of disease. | Vesicles are formed on first and second day of disease. |
| Vesicles are multilocular and are not emptied by single prick of pin. | Vesicles are unilocular and are completely emptied by single prick of pin. |
| Eruption becomes pustular on sixth and seventh days of disease. | Eruption has completely disappeared by seventh or eighth day of disease. |
| Umbilication is usually most pronounced about eighth day, but may not be present at all. | In *all cases* umbilication never occurs in the single vesicle, but an appearance like it is sometimes seen at the junction of coalescent vesicles. |
| Dessication begins on eighth to twelfth day, and is complete in three or four weeks. | Dessication may begin on second day and is, in most cases, entirely completed before eighth day. |

| | |
|---|---|
| The eruption of smallpox in the pustular stage is characteristic and unlike anything else. | A typical pustule is never seen in chicken pox. |
| There is no disease known as Cuban itch that resembles smallpox. Eruptions in Cuba, known as Cuban itch, are ring-worm and prickly heat. | The eruption in empetigo contagiosa consists of a raised surface, surmounted by many very small vesicles. |
| The smallpox papule is surmounted by a single vesicle. | The disease does not resemble smallpox at all. |

State Health Officer Blunt is now on duty again, after having experienced an attack of pneumonia at Galveston.

Mississippi has just enacted a law providing for compulsory vaccination, and providing free virus, the State and county each paying one-half the cost of the virus. Texas should do likewise.

